# Deep-Sea *In Situ* Insights into the Formation of Zero-Valent Sulfur Driven by a Bacterial Thiosulfate Oxidation Pathway

**DOI:** 10.1128/mbio.00143-22

**Published:** 2022-07-19

**Authors:** Ruining Cai, Wanying He, Rui Liu, Jing Zhang, Xin Zhang, Chaomin Sun

**Affiliations:** a CAS Key Laboratory of Experimental Marine Biology & Center of Deep Sea Research, Institute of Oceanology, Chinese Academy of Sciences, Qingdao, China; b Laboratory for Marine Biology and Biotechnology, Qingdao National Laboratory for Marine Science and Technology, Qingdao, China; c College of Earth Science, University of Chinese Academy of Sciences, Beijing, China; d CAS Key Laboratory of Marine Geology and Environment & Center of Deep Sea Research, Institute of Oceanology, Chinese Academy of Sciences, Qingdao, China; e Center of Ocean Mega-Science, Chinese Academy of Sciences, Qingdao, China; Georgia Institute of Technology; University of Georgia

**Keywords:** zero-valent sulfur, deep sea, cold seep, *in situ*, thiosulfate oxidation

## Abstract

Zero-valent sulfur (ZVS) distributes widely in the deep-sea cold seep, which is an important immediate in the sulfur cycle of cold seep. In our previous work, we described a novel thiosulfate oxidation pathway determined by thiosulfate dehydrogenase (TsdA) and thiosulfohydrolase (SoxB) mediating the conversion of thiosulfate to ZVS in the deep-sea cold seep bacterium Erythrobacter flavus 21-3. However, the occurrence and ecological role of this pathway in the deep-sea cold seep were obscure. Here, we cultured E. flavus 21-3 in the deep-sea cold seep for 10 days and demonstrated its capability of forming ZVS in the *in situ* field. Based on proteomic, stoichiometric analyses and microscopic observation, we found that this thiosulfate oxidation pathway benefited E. flavus 21-3 to adapt the cold seep conditions. Notably, ~25% metagenomes assembled genomes derived from the shallow sediments of cold seeps contained both *tsdA* and *soxB*, where presented abundant sulfur metabolism-related genes and active sulfur cycle. Our results suggested that the thiosulfate oxidation pathway determined by TsdA and SoxB existed across many bacteria inhabiting in the cold seep and frequently used by microbes to take part in the active cold seep biogeochemical sulfur cycle.

## INTRODUCTION

Zero-valent sulfur (ZVS) distributes widely in the deep-sea cold seep, which is an important immediate in the active sulfur cycle of the cold seep (1–3). ZVS production for bacteria is a strategy to conserve energy (4). Thiosulfate is regarded as a key substance in the sulfur cycle of marine sediments, which is a common substrate oxidized by almost all sulfur bacteria (5). Therefore, microbial thiosulfate oxidation pathways provide a new clue to the ZVS formation in the deep-sea cold seep (6, 7).

The conversion of thiosulfate to ZVS can be completed by microbes through three pathways at least. For the typical sulfur-oxidizing enzyme (Sox) system, the multienzyme complex consisting of SoxXA, SoxYZ, SoxB, and SoxCD has the capacity of oxidizing sulfide, ZVS, sulfite, and thiosulfate to sulfate as the final product (8, 9). This pathway operates in photo- and chemolithotrophic *Alphaproteobacteria* (9, 10). However, for organisms deficient in SoxCD complex, the sulfur atom of sulfane bound to SoxY cannot be oxidized further, whereas it is transferred to produce periplasmic or extracellular ZVS (11–14). Exceptionally, Thiomicrospira thermophila could produce extracellular ZVS at low pH though *soxCD* genes (15). Tetrathionate intermediate (S_4_I) pathway is also a thiosulfate oxidation pathway distributing in *Proteobacteria* including *Acidithiobacillia*, *Alpha-*, *Beta-*, *and Gamma proteobacteria* (16–19). This pathway was made up of thiosulfate: quinol oxidoreductase (TQO or DoxDA) and tetrathionate hydrolase (TetH or TTH) (20). TQO oxidizes thiosulfate to tetrathionate while TTH hydrolyzes tetrathionate to thiosulfate, ZVS and sulfate as final products (19, 21).

In our recent work, a novel thiosulfate oxidation pathway discovered in the deep-sea cold seep bacterium Erythrobacter flavus 21-3 was described, which provided a new clue about the formation of ZVS (6). Thiosulfate dehydrogenase (TsdA) and thiosulfohydrolase (SoxB) were identified to play key roles in the conversion of thiosulfate to ZVS. In this novel pathway, TsdA converted thiosulfate to tetrathionate, and SoxB liberated sulfone from tetrathionate to form ZVS. However, whether this novel thiosulfate oxidation pathway occurs in the deep-sea cold seep remains obscure. Actually, key genes involved in this novel pathway were found in many sulfur oxidizing bacteria living in the deep-sea cold seep (6, 22). Therefore, microbes using this pathway were proposed to be an important part in the sulfur cycle of deep-sea cold seeps (6). However, because of huge differences between laboratory and *in situ* environment, whether bacteria perform the same thiosulfate oxidation pathway in the deep-sea condition as they were cultivated in the laboratory should be further confirmed.

To investigate whether E. flavus 21-3 produces ZVS in the deep-sea cold seep using the same pathway, we *in situ* incubated E. flavus 21-3 wild type (WT) and mutants with deletion of key gene(s) determining the formation of ZVS in the deep-sea cold seep located in the South China Sea for 10 days, and we found E. flavus 21-3 could produce ZVS in the deep-sea cold seep through the same thiosulfate oxidation pathway. Based on proteomic, stoichiometric, and microscopic results, distinctions between E. flavus 21-3 cultivated in the deep-sea cold seep and laboratory with/without activating the thiosulfate oxidation pathway were compared. Moreover, sulfur metabolism-related genes in the deep-sea cold seep sediments and broad distribution of bacteria potentially used this pathway were investigated and discussed.

## RESULTS AND DISCUSSION

### *E. flavus* 21-3 is capable of producing ZVS in the deep-sea cold seep.

In our recent work (6), in the laboratorial condition, we isolated a bacterium named E. flavus 21-3 from deep-sea cold seep sediments and identified a novel thiosulfate oxidation pathway. It is able to convert thiosulfate to ZVS through thiosulfate dehydrogenase (TsdA) and thiosulfohydrolase (SoxB). Given the special environmental condition of deep-sea cold seep, we sought to ask whether *E. flavus* 21-3 produces ZVS in the cold seep through this pathway. To this end, *E. flavus* 21-3 WT and mutants Δ*soxB* and Δ*tsdA* were *in situ* incubated for 10 days in the cold seep of the South China Sea where we isolated E. flavus 21-3 (Fig. S1 in the supplemental material). To check whether general sulfur-containing substrates involved in sulfur metabolism existed in the study site, the concentrations of sulfide, sulfite, sulfate and thiosulfate in sediments and seawater were measured (Table S1). Among them, thiosulfate, which was regarded as the main substrate driving the formation of ZVS in E. flavus 21-3, was determined as 137.76 μM in sediments. It provided basis for E. flavus 21-3 to perform thiosulfate oxidation in the deep-sea cold seep.

10.1128/mbio.00143-22.2 FIG S1 *In situ* photos of the study process and site. Download FIG S1, DOCX file, 2.0 MB. Copyright © 2022 Cai et al. 2022 Cai et al. https://creativecommons.org/licenses/by/4.0/ This content is distributed under the terms of the Creative Commons Attribution 4.0 International license.

10.1128/mbio.00143-22.5 TABLE S1 The concentrations of sulfide, thiosulfate, sulfate and sulfite present in the seawater and sediments of the study sites. Download Table S1, DOCX file, 0.01 MB. Copyright © 2022 Cai et al. 2022 Cai et al. https://creativecommons.org/licenses/by/4.0/ This content is distributed under the terms of the Creative Commons Attribution 4.0 International license.

**FIG 1:**
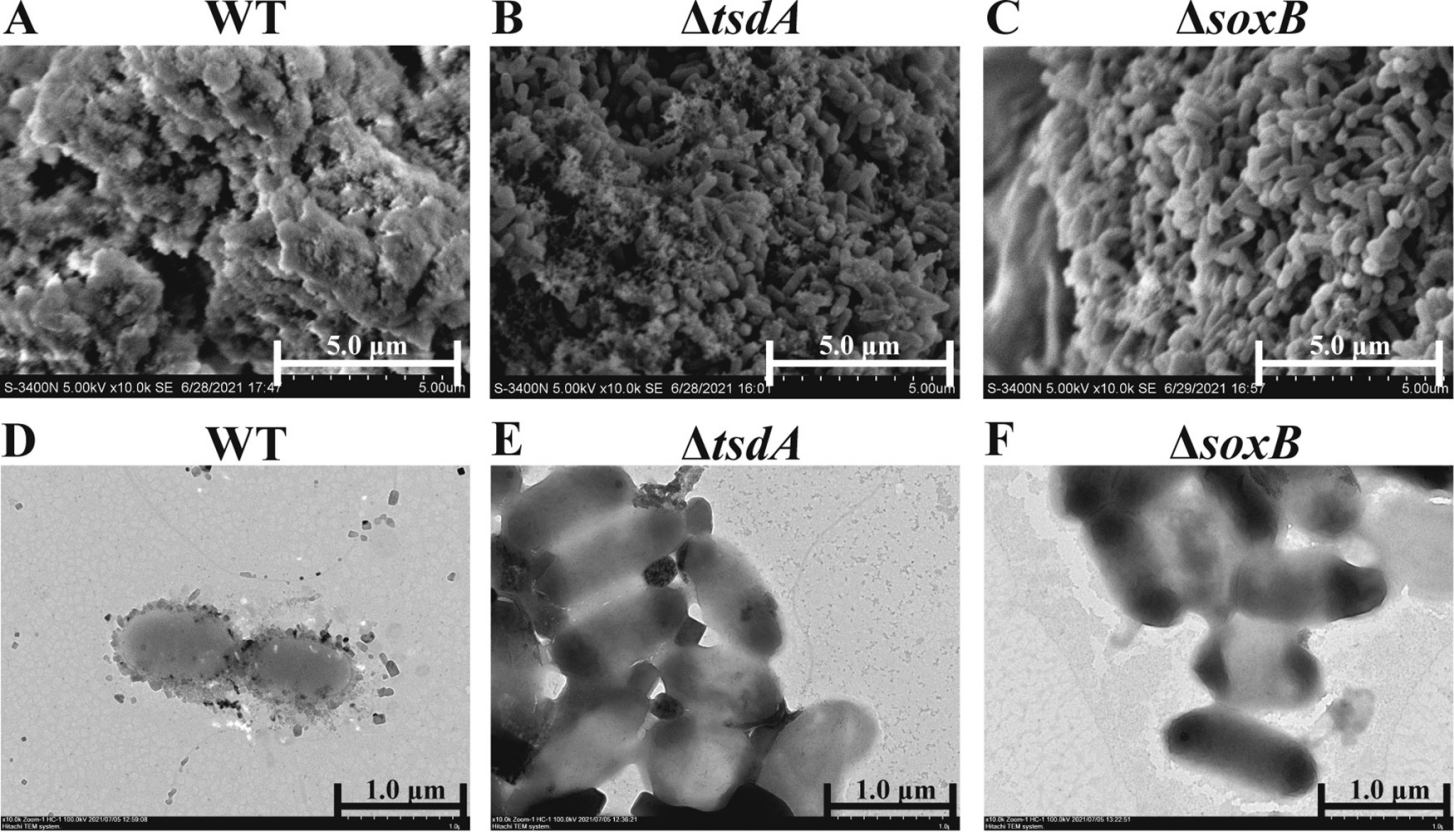
The electron microscopic images of *E. flavus* 21-3 wild type and mutants Δ*tsdA* and Δ*soxB* after 10-day *in situ* cultivation. Scanning electron microscopic (SEM) images of *E. flavus* 21-3 wild type (A) and mutants Δ*tsdA* (B) and Δ*soxB* (C) after 10-day *in situ* cultivation. Transmission electron microscopic (TEM) images of *E. flavus* 21-3 wild type (D) and mutants Δ*tsdA* (E) and Δ*soxB* (F) after 10-day *in situ* cultivation.

**FIG 2:**
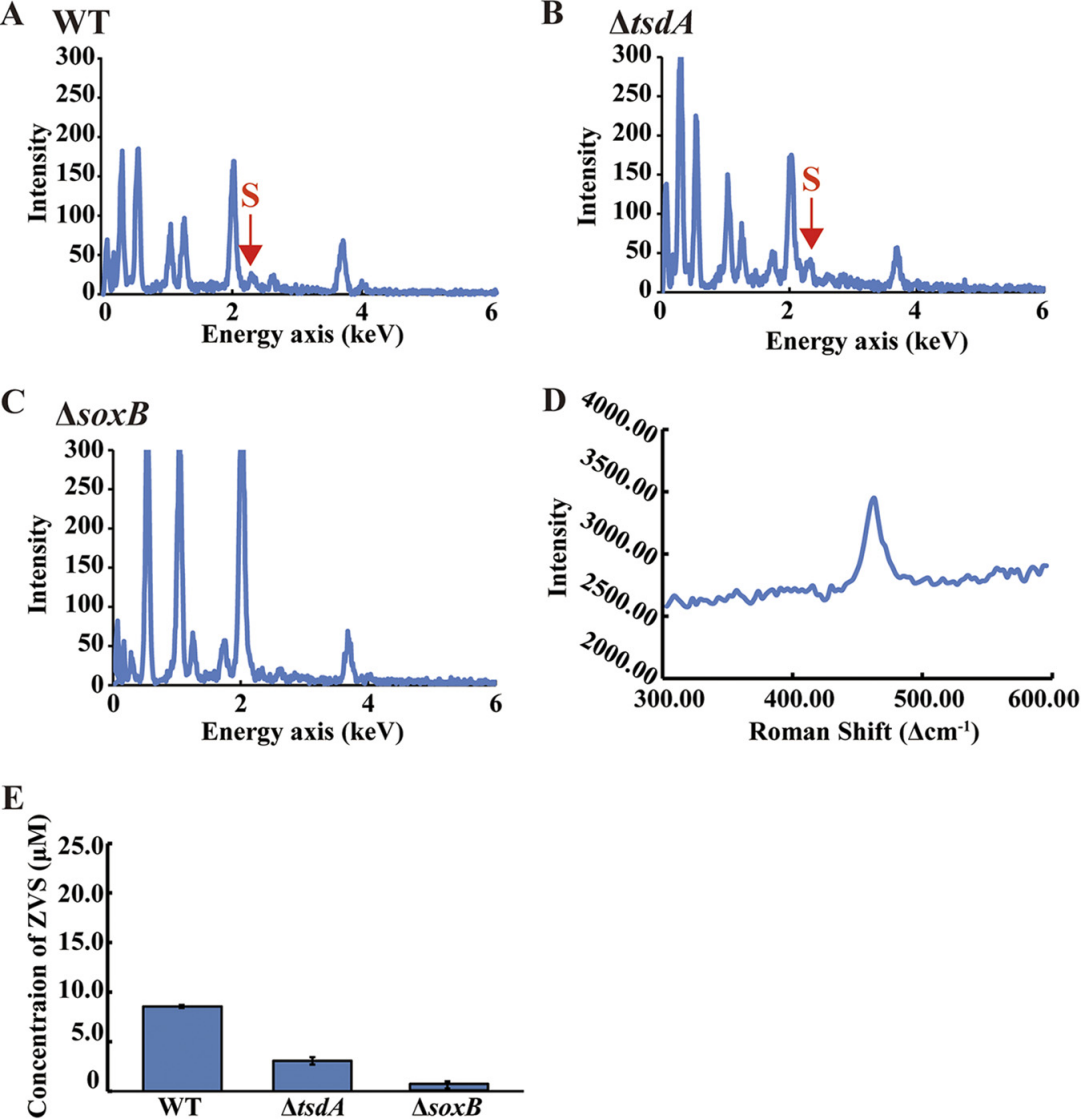
Verification of zero-valent sulfur (ZVS) formed by *E. flavus* 21-3 and mutants Δ*tsdA* and Δ*soxB* that cultured in the deep-sea cold seep. Energy dispersive spectrum analysis of substances formed in the surfaces of *E. flavus* 21-3 wild type (A) and mutants Δ*tsdA* (B) and Δ*soxB* (C). (D) The Raman peak at ~475 Δcm^−1^ of standard S_8_. (E) Measurement of the concentration of ZVS formed by *E. flavus* 21-3 wild type and mutants Δ*tsdA* and Δ*soxB* that cultured in the deep sea for 10 days.

To confirm whether E. flavus 21-3 WT and mutants Δ*soxB* and Δ*tsdA* produced ZVS in the deep-sea cold seep, electron microscopic observation and Roman Spectra analyses were performed. Scanning electron microscopy (SEM) results showed that the WT cells were completely embedded in ample fibrous substances (Fig. 1A). However, less fibrous substances appeared on the surface of the mutant Δ*tsdA* (Fig. 1B), and were even totally absent on the surface of the mutant Δ*soxB* (Fig. 1C). Consistently, TEM results showed that many particles (~100-200 nm) attached to the surfaces of WT (Fig. 1D and Fig. S2A in the supplemental material) and the mutant Δ*tsdA* (Fig. 1E and Fig. S2B) but not to the surface of the mutant Δ*soxB* (Fig. 1F and Fig. S2C). The attachments presented on the surfaces of WT and the mutant Δ*tsdA* were further identified as sulfur-containing substances by EDS (Fig. 2A and B). Consistently, sulfur element couldn’t be detected on the surface of the mutant Δ*soxB* (Fig. 2C). To verify whether these substances attached to the cells included ZVS, the attachments presented on the surface of deep-sea *in situ* cultured cells were extracted and checked by Raman Spectra. The strong Raman peak at ~475 Δcm^−1^ indicated S_8_ (a form of ZVS) was produced by the WT and the mutant Δ*tsdA* in the cold seep (Fig. 2D). Consistently, the highest concentration of ZVS was detected in WT, much less in the mutant Δ*tsdA*, and almost none in the mutant Δ*soxB* (Fig. 2E). Notably, we observed the formation of S_8_ in the deep-sea cold seep where strain 21-3 was isolated (3), suggesting deep-sea microorganisms (e.g., strain 21-3) have potential contribution to the formation of ZVS in deep-sea cold seeps. These results clearly showed that E. flavus 21-3 was able to produce ZVS in the cold seep and *tsdA* and *soxB* were the key genes in determining the formation of ZVS under both *in situ* and laboratorial conditions (6).

10.1128/mbio.00143-22.3 FIG S2 TEM images of embedded and thin sectioned cells of *E. flavus* 21-3 wild type and the mutants Δ*tsdA* and Δ*soxB* after 10-day *in situ* incubation. Download FIG S2, DOCX file, 0.4 MB. Copyright © 2022 Cai et al. 2022 Cai et al. https://creativecommons.org/licenses/by/4.0/ This content is distributed under the terms of the Creative Commons Attribution 4.0 International license.

Besides, membrane vesicle-like structures were also observed on the surfaces of the WT (Fig. S2D) and the mutant Δ*tsdA* (Fig. S2E) but not on the surface of the mutant Δ*soxB* (Fig. S2F) based on the observation of ultrathin sections. Similar structure was observed and regarded as part of the detoxification mechanism in archaea and bacteria (23–25). For example, Thermococcus prieurii used membrane vesicles to export intracellular ZVS for avoiding the toxicity of sulfur in high concentration (23); Allochromatium vinosum, whose membrane vesicles were observed ever, could take up ZVS as electron donor (26, 27); Chlorobaculum tepidum which producing extracellular ZVS through same vesicles, could also transiently attach to sulfur globules for fuel subsequent growth (28, 29). Therefore, the membrane vesicles were proposed to be essential for transportation, storage and utilization of ZVS for microorganisms including E. flavus 21-3. It needs further verification in the future.

### Biological functions of ZVS formation for *E. flavus* 21-3.

**FIG 3:**
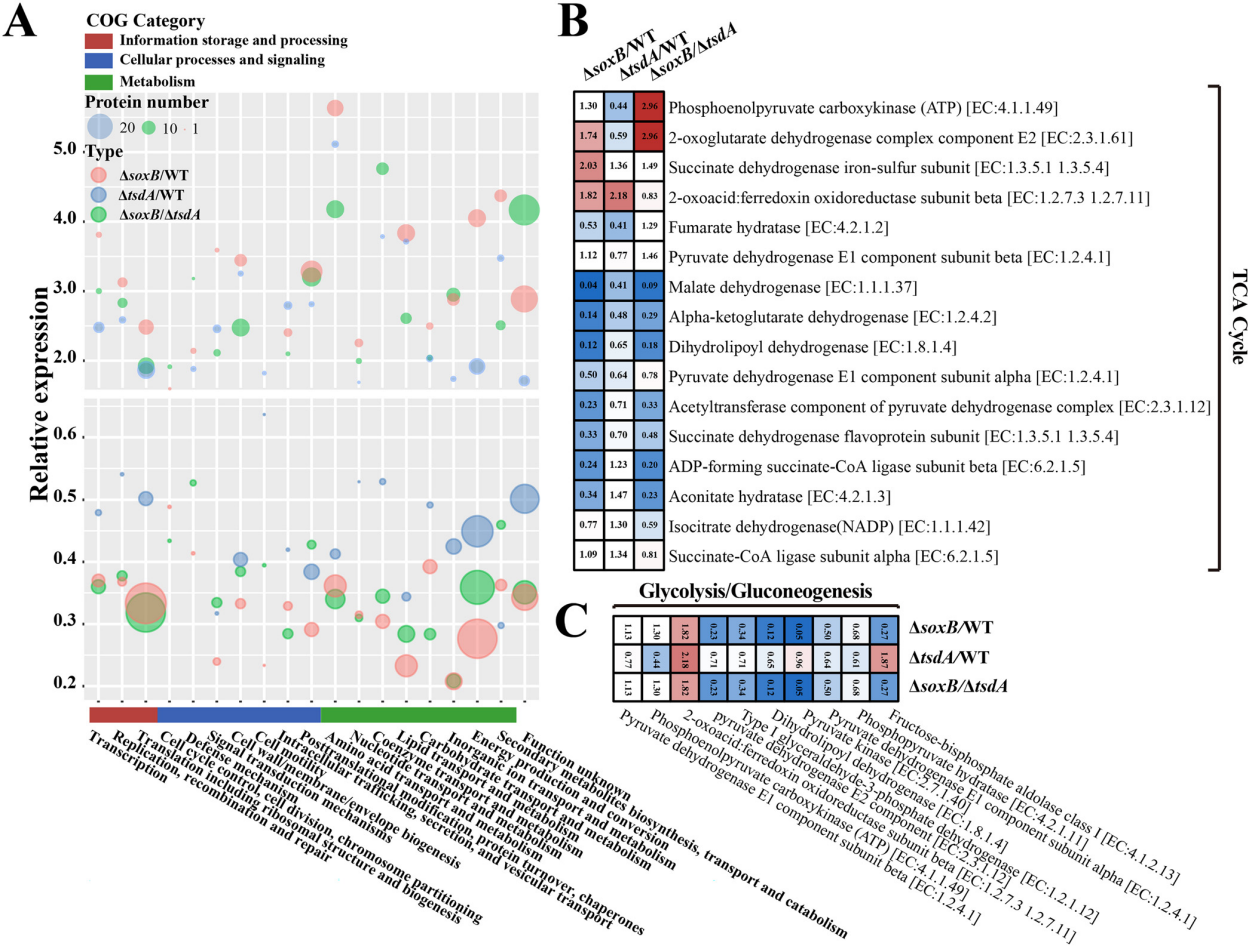
Comparative proteomic analysis of *E. flavus* 21-3 wild type and mutants Δ*tsdA* and Δ*soxB* that cultured in the deep-sea colds seep for 10 days. (A) Comparative analysis of the number and relative expression level of proteins in *E. flavus* 21-3 wild type and mutants Δ*tsdA* and Δ*soxB* in different COG categories. (B) Heatmap analysis of differentially expressed proteins in TCA cycle of *E. flavus* 21-3 wild type and mutants Δ*tsdA* and Δ*soxB*. (C) Heatmap analysis of differentially expressed proteins in glycolysis and gluconeogenesis of *E. flavus* 21-3 wild type and mutants Δ*tsdA* and Δ*soxB*.

Next, we sought to ask what biological functions of ZVS formation for E. flavus 21-3 in the deep-sea cold seep. For this purpose, proteomic assays of *in situ* cultured E. flavus 21-3 WT and mutants (Δ*tsdA* and Δ*soxB*) were performed. Compared with proteins expression of the WT, 126 of 988 and 200 of 850 proteins were significantly down-regulated in the mutants Δ*tsdA* and Δ*soxB*, respectively (*P < *0.05). The abundance-reduced proteins in both mutants Δ*tsdA* and Δ*soxB* fell into the COG categories of energy production and conversion (Fig. 3A). Furthermore, analyses based on KEGG database showed most proteins associated with the glycolysis, gluconeogenesis and TCA cycle were down-regulated in the mutants Δ*tsdA* and Δ*soxB* (Fig. 3B and C). Besides, compared to the mutant Δ*tsdA* which could produce ZVS in the cold seep, most proteins in the mutant Δ*soxB* which couldn’t produce ZVS were down-regulated (Fig. 3). According to these results, we speculate that ZVS should be important to the growth, energy conservation of *in situ* incubated E. flavus 21-3 in the cold seep. E. flavus 21-3 was thus proposed to grow better in the presence of ZVS.

**FIG 4:**
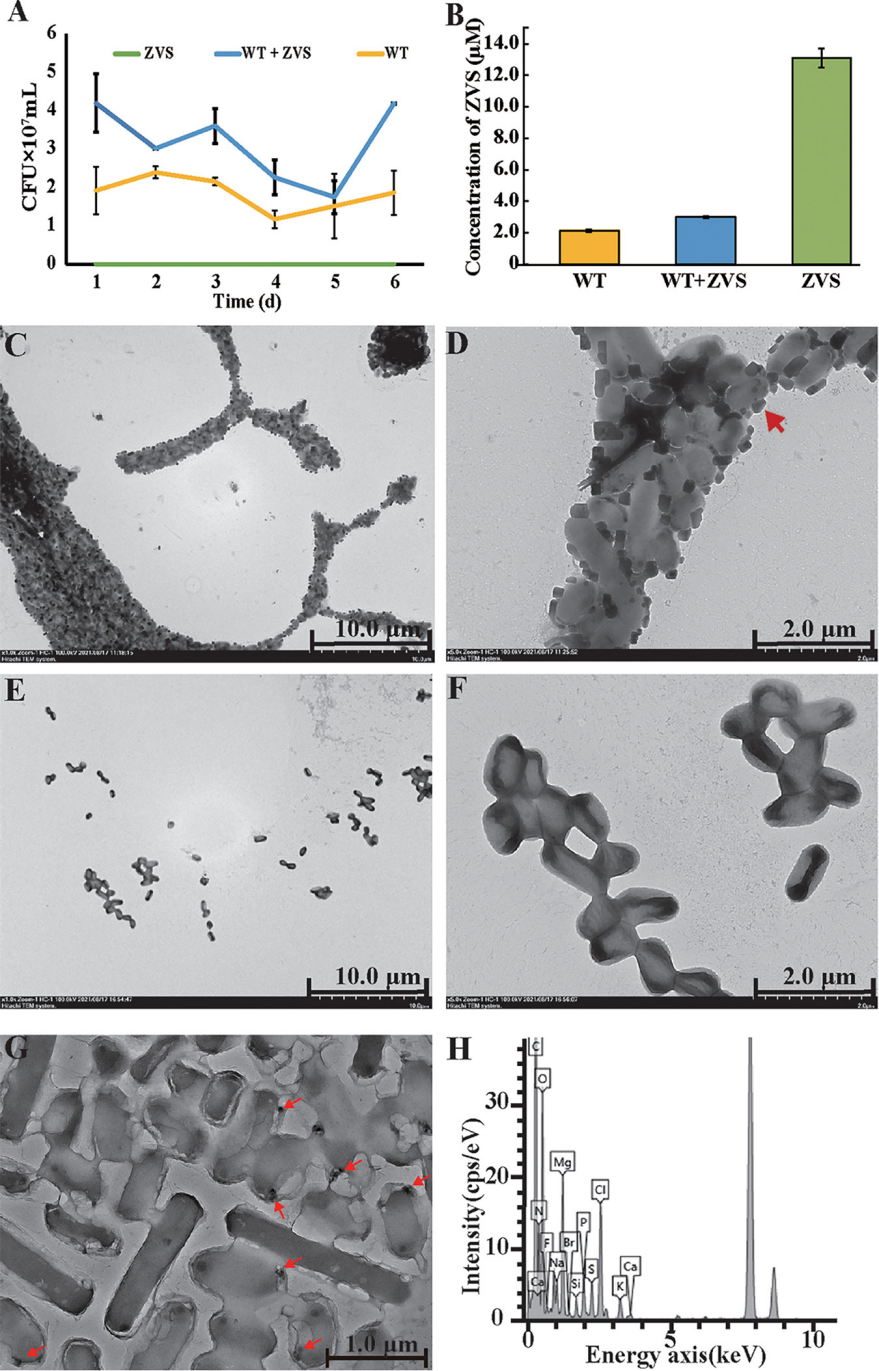
Assay of the effect of ZVS on the growth of *E. flavus* 21-3. (A) Growth curve of *E. flavus* 21-3 that cultivated in the medium supplemented with or without 20 mM biogenic ZVS. (B) Measurement of the concentration of ZVS in *E. flavus* 21-3 that cultured in the medium supplemented with or without 20 mM biogenic ZVS for 6 days. TEM images of *E. flavus* 21-3 that cultivated in the medium supplemented with (C, D) or without (E, F) 20 mM biogenic ZVS for 14 days. (G) TEM images of the substances formed on the surface of *E. flavus* 21-3 that cultivated in the medium supplemented with 20 mM biogenic ZVS for 14 days. (H) Energy dispersive spectrum analysis of the black particles attaching to the cell surface shown in panel G.

To verify the deduction above, ZVS produced by E. flavus 21-3 was purified and added to the ASW as the sole electron donor. Continuous measurements of the biomass showed that ZVS promoted the growth of *E. flavus* 21-3 (Fig. 4A). Accordingly, after 6-day cultivation, the concentration of remaining ZVS significantly decreased, strongly suggesting ZVS was consumed by E. flavus 21-3 (Fig. 4B). To better characterize the growth of E. flavus 21-3 in the medium supplemented without or with ZVS, we further checked the morphology of E. flavus 21-3 that cultured in the above conditions using TEM. The results clearly showed that many extra particles (~200 nm) attached to surfaces of bacterial cells that cultured with supplemental ZVS (Fig. 4C and D), while none of above particles was observed around bacterial cells that cultured without ZVS (Fig. 4E and F). And these particles around bacterial cells were further identified as element sulfur through the EDS analysis (Fig. 4G and H). Of note, the amount of ZVS attached to bacterial cells showed an evident decrease trend along with the incubation time from 7 days (Fig. S3A and S3B) to 14 days (Fig. S3C and S3D), strongly suggesting E. flavus 21-3 consumed ZVS as nutrient and thereby generating energy to support bacterial growth.

10.1128/mbio.00143-22.4 FIG S3 TEM images of wild type *E. flavus* 21-3 that cultivated with sulfur globules as the sole electron donor after 7 days and 14 days. Download FIG S3, DOCX file, 1.6 MB. Copyright © 2022 Cai et al. 2022 Cai et al. https://creativecommons.org/licenses/by/4.0/ This content is distributed under the terms of the Creative Commons Attribution 4.0 International license.

In combination of above results, we proposed that ZVS production and secretion were energy conservation and detoxification strategies for E. flavus 21-3 to adapt deep-sea cold seeps. They utilized abundant thiosulfate as nutrition, converted it to ZVS. Then ZVS was transported outside of cells for avoiding accumulating high concentration of intracellular sulfur. Subsequently, as their energy reserves, E. flavus 21-3 attached to and utilized ZVS as the electron donor for better growth.

### Comparative analyses of ZVS formation of *E. flavus* 21-3 incubated in the deep-sea cold seep and laboratory.

**FIG 5:**
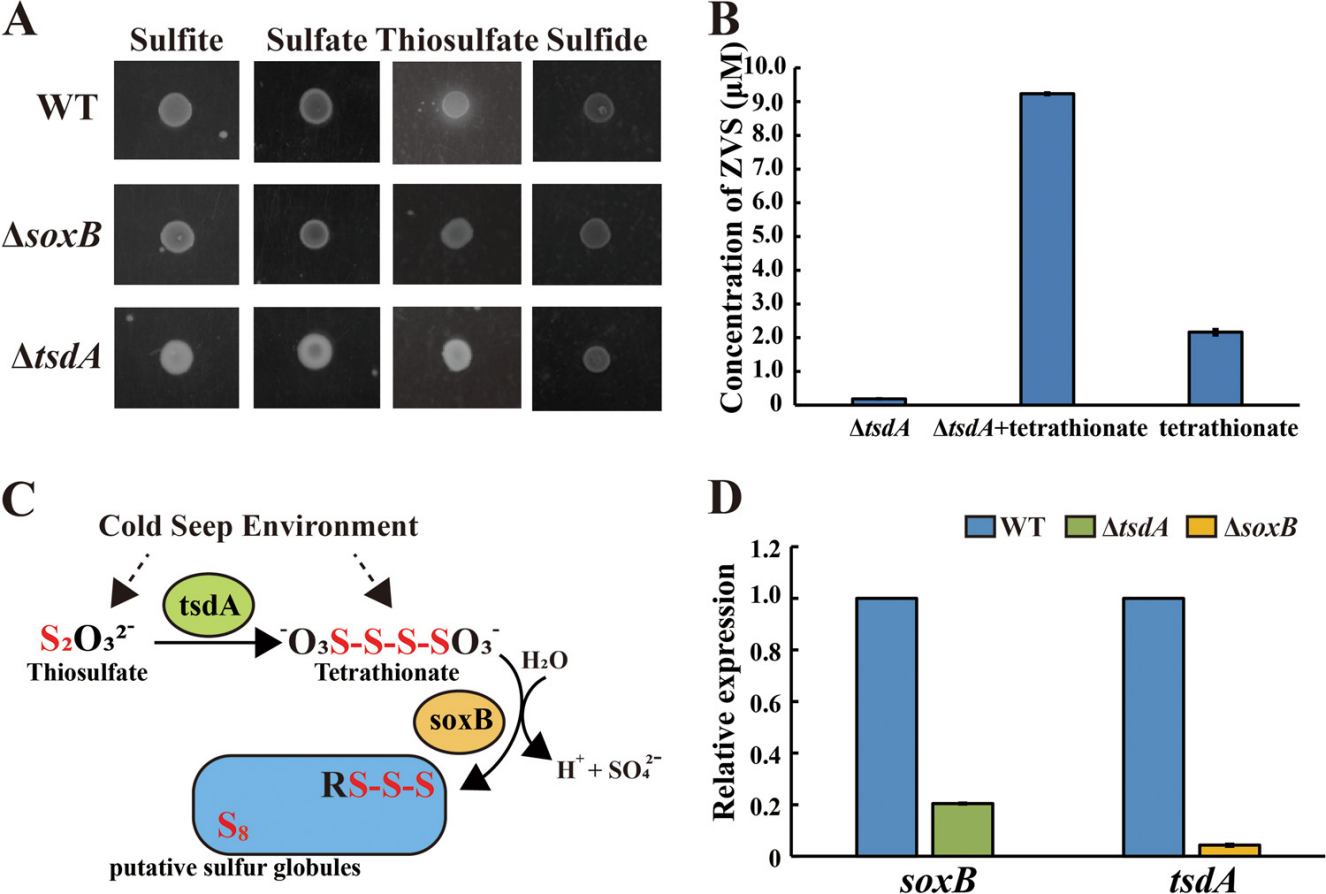
Tetrathionate contributes to ZVS formation of *E. flavus* 21-3 that cultured in the deep-sea cold seep. (A) Analysis of the formation of ZVS by *E. flavus* 21-3 wild type and mutants Δ*tsdA* and Δ*soxB* in the laboratorial condition in the medium supplemented with 5 mM sulfide, 40 mM sulfate, 40 mM sulfite or 20 mM thiosulfate. (B) Measurement of the yield of ZVS formed by the mutant Δ*tsdA* that cultivated in the medium supplemented with or without 10 mM tetrathionate. (C) The proposed thiosulfate oxidation pathway of *E. flavus* 21-3 cultured in the deep-sea cold seep. (D) Relative gene expression of *tsdA and soxB* in the mutants Δ*soxB* and Δ*tsdA* that cultured in the deep-sea cold seep.

In our previous study (6), we found that the mutant Δ*tsdA* was unable to produce ZVS in the presence of thiosulfate in the laboratorial condition due to the defect of conversion of thiosulfate to tetrathionate. However, the mutant Δ*tsdA* was able to produce a small amount of ZVS in the deep-sea cold seep (Fig. 1 and 2). Given the existence of different sulfur-containing compounds in the cold seep (Table S1 in the supplemental material), we thus asked whether the mutant Δ*tsdA* could utilize other substrates to produce ZVS. Accordingly, the mutant Δ*tsdA* was incubated in the medium supplemented with 5 mM sulfide, 40 mM sulfate, 40 mM sulfite, 20 mM thiosulfate or 10 mM tetrathionate, and ZVS was only produced in the sterile seawater added with tetrathionate (Fig. 5A and B). Therefore, we proposed that the mutant Δ*tsdA* was able to take in tetrathionate from the cold seep and thereby transferring to ZVS (Fig. 5C). To further verify this deduction, the expressions of *soxB* and *tsdA* in the WT and the mutant Δ*tsdA* or Δ*soxB* that incubated in the deep-sea cold seep were measured through qRT-PCR. Transcription of *soxB* in the mutant Δ*tsdA* could be detected, while about 5-fold lower than that in the WT (Fig. 5D). On the other hand, transcription of *tsdA* in the mutant Δ*soxB* was hardly detected (Fig. 5D). Therefore, SoxB was proposed to be functional of metabolizing tetrathionate in the mutant Δ*tsdA*. But the efficiency of ZVS production was much lower than the WT based on the yield of ZVS produced by the mutant Δ*tsdA* was less than the WT (Fig. 2E). We ever tried to determine the concentration of tetrathionate in the cold seep sediments but failed because tetrathionate was unstable in the sediments when transported and stored (7, 30). However, several reports described that tetrathionate was an important immediate of the sulfur cycle in marine sediments (31–33). Therefore, we proposed tetrathionate should exist in the deep-sea cold seep where we isolated strain 21-3 and performed *in situ* experiments. Though the formation of ZVS in the mutant Δ*tsdA* was a bit different for *in situ* and laboratorial conditions, the thiosulfate oxidation pathway determined by *tsdA* and *soxB* worked in both deep-sea and laboratorial conditions.

**FIG 6:**
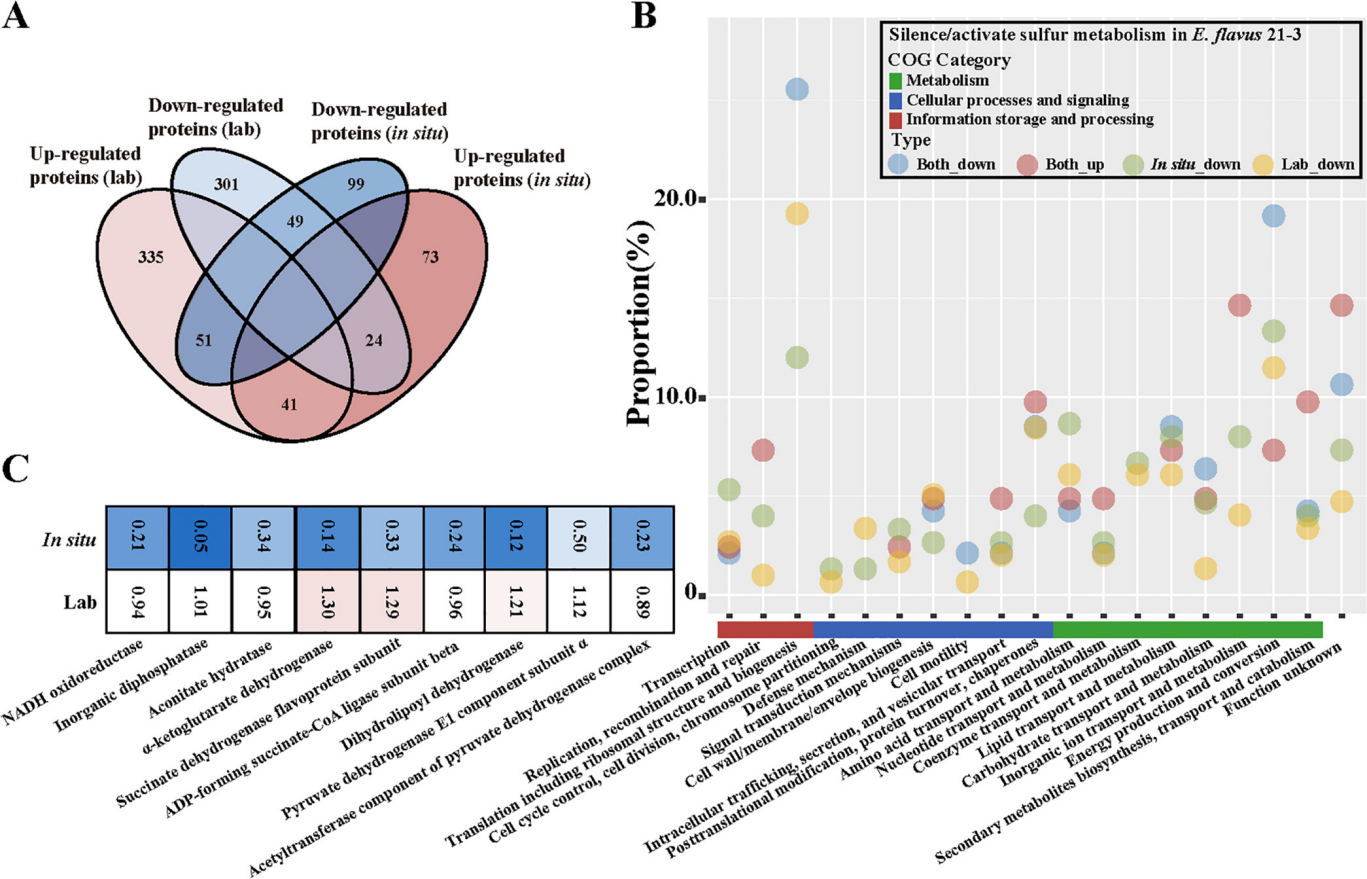
Comparative proteomic analysis of *E. flavus* 21-3 wild type cultivated in the laboratory or cold seep (*in situ*) when activating or silencing the thiosulfate oxidation pathway (A) Venn diagram depicting unique and shared orthologous protein clusters in each of four samples (up-regulated/down-regulated proteins in the *E. flavus* 21-3 wild type cultivated in the laboratory or deep-sea cold seep). (B) The proportion of expressed proteins in *E. flavus* 21-3 in different COG categories. The expressed proteins were indicated as proteins which were both up-regulated or both down-regulated in the laboratory and cold seeps, or were down-regulated in either of ones. (C) Heatmap analysis of differentially expressed proteins contributing to energy production and conversion of *E. flavus* 21-3 that cultivated in the laboratory and deep-sea cold seep.

From above results, because of totally different conditions between laboratory and deep-sea cold seep, laboratorial study cannot truly show how bacteria work in *in situ* environment. So, to further compare the metabolism especially the thiosulfate oxidation pathway in E. flavus 21-3 incubated in the cold seep and laboratory, comparative proteomic analyses were performed. Based on results above, when *soxB* was knocked out, the thiosulfate oxidation pathway of E. flavus 21-3 was silenced and ZVS was absent. When we compared the protein expressions between WT and the mutant Δ*soxB* cultivated in the cold seep and laboratory respectively, big differences were found. Three hundred and one of 807 and 99 of 337 proteins were down-regulated in E. flavus 21-3 that incubated respectively in the laboratory and cold seep when the thiosulfate oxidation pathway was silenced (Fig. 6A). And only 49 proteins were down-regulated in both two conditions described above. The functional composition of unique and shared up-regulated/down-regulated proteins of *in situ* and laboratory groups was further analyzed based on the COG categories. The result showed that shared down-regulated proteins majorly contributed to energy production and conversion and translation including ribosome structure and biogenesis (Fig. 6B). *In situ* unique down-regulated proteins mostly belonged to energy production and conversion and contributed to energy production and conversion showed few changes in ones expressed in the laboratory (Fig. 6C). It meant that the presence of the thiosulfate oxidation pathway was more important for *in situ* incubated E. flavus 21-3 than those cultivated in the laboratory. Besides, in 13 of 19 COG categories, unique downregulated proteins of *in situ* incubated *E. flavus* 21-3 were more than those incubated in the laboratory, suggesting that the absence of the thiosulfate oxidation pathway impacted metabolisms of *in situ* incubated E. flavus 21-3 more negatively. These results highlighted the importance of the thiosulfate oxidation pathway for E. flavus 21-3 to adapt deep-sea special conditions.

### Broad distribution of the novel thiosulfate oxidation pathway in deep-sea cold seeps.

**FIG 7:**
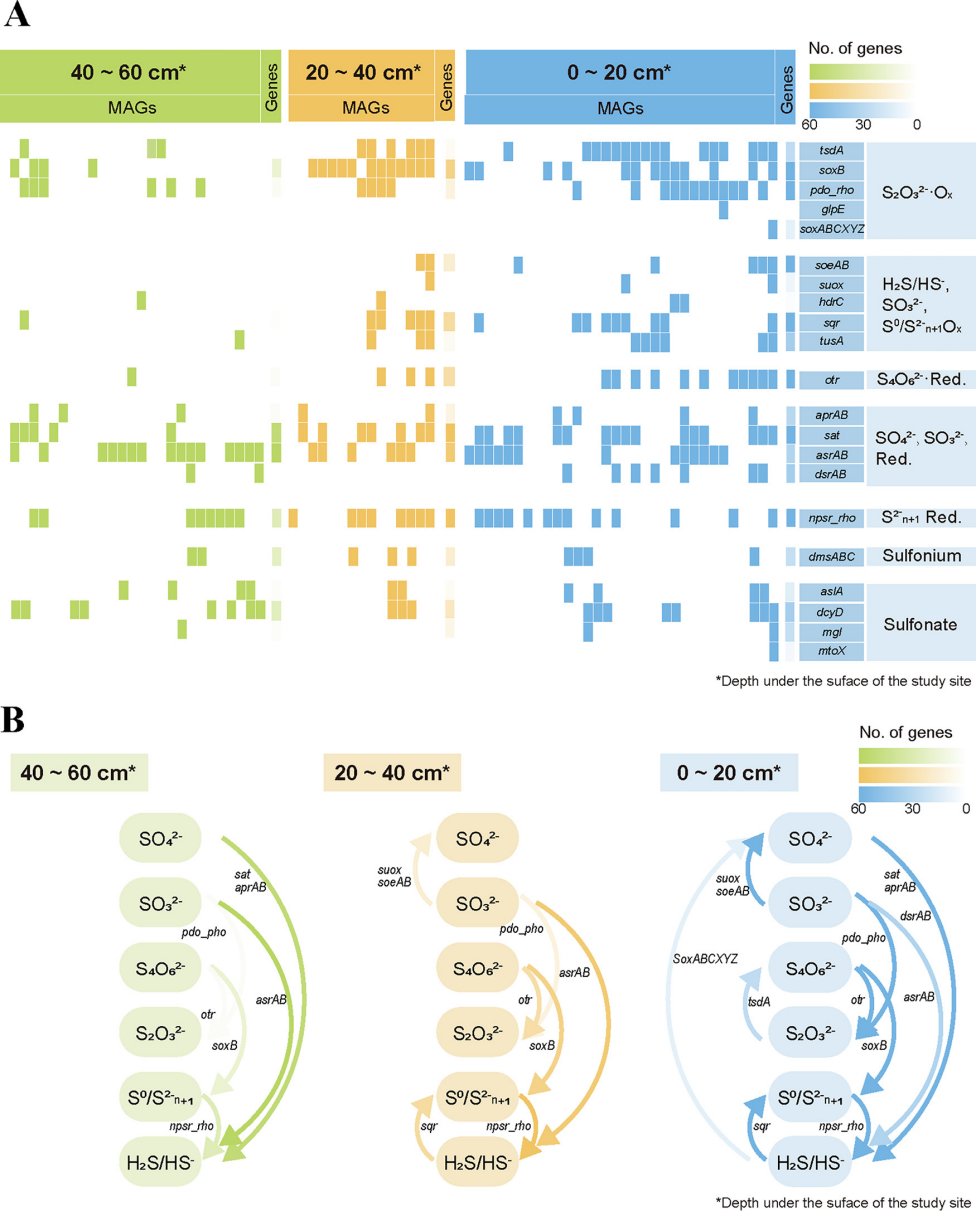
Metagenomic analysis of the distribution of sulfur metabolism-related genes in different depths of the study site. (A) Vertical profiles of sulfur cycling associated genes identified in metagenomes along geochemical gradients. The numbers of genes per sample were normalized. The genes used in this analysis were listed in Table S3. (B) Schematic representation of the sulfur cycle in different depths of the study site.

To investigate the sulfur cycle of cold seep sediments of the study site, metagenomic analyses were performed. Sediments sampled for sequencing were collected from different depths: 0–20 cm, 20–40 cm, and 40–60 cm below the seafloor; and 2,653,537 gene sequences and 81 bacterial metagenomes sequenced from sediments were annotated based on the KEGG and Uniprot database. The result showed that the abundance of genes associated with sulfur cycle decreased with the increasing depth of sediments (Fig. 7A). Proposed sulfur cycles in the sediments of different depths were constructed based on the distribution of metagenes (Fig. 7B). Sulfur oxidation genes were decreased with increasing depth. The presence of sulfur oxidation resulted in more abundant sulfur cycling associated genes in the shallower sediments.

10.1128/mbio.00143-22.7 TABLE S3 Sulfur metabolism-related genes used in metagenomic analysis. Download Table S3, DOCX file, 0.02 MB. Copyright © 2022 Cai et al. 2022 Cai et al. https://creativecommons.org/licenses/by/4.0/ This content is distributed under the terms of the Creative Commons Attribution 4.0 International license.

To further investigate the contribution of the thiosulfate oxidation pathway identified in E. flavus 21-3 in the cold seep, *soxB* and *tsdA* gene homologs were identified in the metagenomic data. In the 0–40 cm sediments, the metagenomes containing both *tsdA* and *soxB* homologous genes constituted ~25% of all bacteria metagenomes. In the 0–20 cm sediments, more *tsdA* and *soxB* homologous genes were identified. It meant that bacteria, which potentially possess the thiosulfate oxidation pathway identified in E. flavus 21-3, were mainly distributed in the shallow cold seep sediments which containing abundant sulfur cycling genes. Given the key roles of *tsdA* and *soxB* gene homologs, this pathway potentially existed in many microorganisms in the cold seep. Besides, abundant ZVS was ever found in the shallow sediments of cold seep and was regarded as important immediate in the active sulfur cycle of cold seep (1–3). Therefore, we proposed that the microbial contribution of this pathway to the formation of ZVS and the sulfur cycle in the cold seep couldn’t be ignored.

## MATERIALS AND METHODS

### *In situ* cultivation of *E. flavus* 21-3.

E. flavus 21-3 WT and mutants with deletion of key gene(s) determining the formation of ZVS were cultivated in 50 mL artificial seawater (ASW) 2216E broth (0.5% tryptone, 0.1% yeast extract in 50 mL ASW) at 28°C with shaking at a speed of 150 rpm until OD_600_ ≈ 0.1. The ASW contained: 24.47 g NaCl, 3.917 g Na_2_SO_4_, 0.664 g KCl, 0.024 g SrCl, 4.981 g MgCl·6H_2_O, 1.102 g CaCl_2_, 0.192 g NaHCO_3_, 0.026 g H_3_BO_4_, and 0.0039 g NaF per 1 L of Milli-Q water. The pH was adjusted to 7.2–7.5 using 1 M NaOH. Cells were collected by centrifugation at 1,000 × *g* for 10 min and washed three times in the ASW. Then washed cells were transferred to 50 mL ASW in the dialysis tubes which allow exchanging ions in the cold seep. Finally, strains were incubated in the cold seep of the South China Sea for 10 days using the remotely operated vehicle (ROV) of RV *KEXUE* as previously described (34). Three biological replicates were performed.

### ZVS purification.

One liter cultures of *E. flavus* 21-3 were cultured at 28°C in the 2216E medium with 40 mM thiosulfate for 24 h. ZVS was purified from cultures by sucrose density gradient centrifugation (28, 35). ZVS and cells were collected by centrifugation at 1,000 × *g* for 10 min at 10°C. Cells and ZVS were resuspended in 5 mL sterile 2.5 M sucrose solution (ρ ≈ 1.32 g/mL) after removing the supernatant. The suspension was transferred into 45 mL of sterile 2.5 M sucrose solution. The ZVS was pelleted through the sucrose solution by centrifugation at 4,000 × *g* for 10 min at 10°C. The supernatant was removed and the collected ZVS was resuspended in 100 mL of sterile 2.5 M sucrose solution two more times. Collected ZVS resuspended with 50 mL sterile ASW was centrifuged at 16,200 × *g* for 5 min at 4°C. This step was repeated twice to remove sucrose. ZVS was resuspended and vortexed for several minutes to detach any remaining cells and allowed settling without centrifugation. The supernatant was removed. The latter step was repeated two more times. Then, the pellets were suspended with 75% alcohol and centrifuged at 5,000 × *g* for 10 min at 4°C. Finally, the supernatant was removed, the pellets were resuspended in sterile seawater and the suspension was stored at 4°C.

### Cultivation of *E. flavus* 21-3 WT and mutants Δ*tsdA* and Δ*soxB* in the medium supplemented with sulfide, sulfate, sulfite, thiosulfate, or ZVS.

To confirm whether E. flavus 21-3 grew better in the presence of ZVS, 20 mM purified ZVS produced by E. flavus 21-3 was added to 50 mL sterilized ASW to cultivate *E. flavus* 21-3 at 28°C with shaking at a speed of 150 rpm for 7 or 14 days; 50 mL ASW added 20 mM ZVS and 50 mL ASW added E. flavus 21-3 were set as control groups. The growth condition was determined by the plate count method. Briefly, medium was diluted gradually, spread on the 2216E medium plate and counted the number of colonies after 3-day cultivation.

To detect whether E. flavus 21-3 WT and mutants Δ*tsdA* and Δ*soxB* could utilize sulfide, sulfate, sulfite and thiosulfate, 2216E solid medium supplemented with 5 mM sodium sulfide, 40 mM sodium sulfate, 40 mM sodium sulfite or 20 mM sodium thiosulfate was used to cultivate E. flavus 21-3 WT and mutants Δ*tsdA* and Δ*soxB*.

To detect whether E. flavus 21-3 mutant Δ*tsdA* transformed tetrathionate to ZVS, the mutant Δ*tsdA* was cultivated in 50 mL sterilized ASW supplemented with 10 mM sodium tetrathionate at 28°C with shaking at a speed of 150 rpm for 3 days; 50 mL sterilized ASW with 10 mM sodium tetrathionate and 50 mL sterilized ASW were set as control groups. The concentration of ZVS was determined as the method described in the following part.

### Electron microscopic analyses of bacterial cells and ZVS produced by *E. flavus* 21-3 in the deep-sea cold seep.

To observe the morphological characteristics of the *in situ* incubated bacteria, cells were collected by centrifugation (1,000 × *g*, 10 min, 4°C), preserved in 25% (vol/vol) glutaraldehyde overnight at 4°C and washed three times using phosphate-buffered saline (PBS) in the next day. Later, samples were dehydrated in the ethanol solution of 30%, 50%, 70%, 90% and 100% for 10 min each time. Then samples were transferred to isoamyl acetate for 20 min at room temperature. Finally, the samples were dried by critical-point drying and coated with graphite and gold. SEM (S-3400N, Hitachi, Japan) was performed to observe samples at 5 keV. For transmission electron microscopy (TEM), *in situ* incubated strains were collected by centrifugation (1,000 × *g*, 10 min, 4°C), washed three times using PBS and dried at room temperature. TEM (HT7700; Hitachi, Japan) was used to observe samples at 100 keV. To identify the element component of cell attachments, Energy-Dispersive Spectrum (EDS) (model 550i, IXRF systems, USA) equipment with SEM was used at an accelerating voltage of 5 keV for 30 s.

To identify the ZVS produced by *E. flavus* 21-3, TEM was used to observe the morphology of ZVS firstly and Raman spectrum (WITec alpha300 R system; WITec Company, Germany) was used to identify the components and structures. After incubation with ZVS, *E. flavus* 21-3 treated as described above was observed through TEM (JEM-2100PLUS, Jeol, Japan). And EDS (X-Max 80. Oxford Instruments, UK) equipment with TEM was used at accelerating voltage to identify the element component of cell attachments.

To further observe the morphological characteristics of bacterial cells, ultrathin-section electron microscopic observation was performed as described previously (36, 37). Briefly, samples were prepared as procedures for SEM observation, and then the dehydrated samples were embedded in a plastic resin. Ultrathin sections (50–70 nm) of cells were prepared with an ultramicrotome (Leica EM UC7, Germany), stained with uranyl acetate and lead citrate. All samples were examined using TEM (HT7700, Hitachi, Japan) at 100 kV.

### Analytical techniques for the determination of sulfate, sulfite, sulfide, thiosulfate and ZVS.

The concentration of sulfate, sulfite and thiosulfate in the seawater and sediment of the study site was monitored by ion chromatography (ECO-IC, Shimadzu, Japan) fitted with a Shodex IC SI-52 4E column (Shodex, Japan) at a constant column temperature of 25°C. Then the column was eluted with 6.0 mM Na_2_CO_3_ and 2.0 mM NaHCO_3_ with a flow of 0.7 mL/min. The concentration of sulfide in the seawater and sediment of the study was determined by iodometric determination (38). Briefly, 10 mL of sample solution (0.5 g sediments dissolved in 10 mL ultra-pure water or 10 mL seawater, all samples were filtered with a pore size of 0.22 μm) was mixed with 25 mL cadmium acetate solution to precipitate sulfide in forms of cadmium sulfide. Then precipitating cadmium sulfide was collected and mixed with 10 mL 0.1 M iodine solution and 3 mL hydrochloric acid. The mixture was titrated with 0.1 M sodium thiosulfate solution. The titration endpoint was determined by an automatic potentiometric titrator (T940, Thermo Fisher Scientific, USA) and the volume of consumed thiosulfate solution was recorded to calculate the concentration of sulfide. ZVS was extracted from the cultured medium using chloroform according to the method described previously (39, 40). Briefly, 3 mL sample was extracted three times using a total of 5 mL chloroform. The extracted chloroform was measured on a UV-Vis spectrometer (Infinite M1000 Pro; Tecan, Männedorf, Switzerland) at 270 nm.

### Quantitative real-time PCR (qRT-PCR).

For qRT-PCR, *in situ* incubated mutants Δ*tsdA* and Δ*soxB* of *E. flavus* 21-3 were collected by centrifugation (10,000 × *g*, 10 min, 4°C). Total RNAs from each sample were extracted using TRIzol reagent (Solarbio, China). The concentration of total RNAs was measured by Spectrophotometer (NanoPhotometer NP80, Implen, Germany). Then RNAs were reverse transcribed into cDNA using ReverTra Ace™ qPCR RT Master Mix with gDNA Remover (TOYOBO, Japan). Transcriptional levels of different genes were determined by qRT-PCR using SYBR Green Realtime PCR Master Mix (TOYOBO, Japan) and the QuantStudio^TM^ 6 Flex (Thermo Fisher Scientific, USA). The condition of PCR was set as following: 95°C for 1 min, followed by 40 cycles of denaturation at 95°C for 15 s, annealing at 55°C for 15 s, and extension at 72°C for 15 s. 16S rRNA was used as an internal reference. The relative gene expression was calculated using the 2^−ΔΔCt^ method with each transcript signal normalized to that of 16S rRNA (41). Primers used were listed in the supplementary information (Table S2 in the supplemental material). All qRT-PCR runs were performed in three biological and three technical replicates.

10.1128/mbio.00143-22.6 TABLE S2 Strains, plasmids and primers used in this study. Download Table S2, DOCX file, 0.02 MB. Copyright © 2022 Cai et al. 2022 Cai et al. https://creativecommons.org/licenses/by/4.0/ This content is distributed under the terms of the Creative Commons Attribution 4.0 International license.

### Proteomic analysis.

For proteomic analysis, *in situ* incubated bacterial cells were collected by centrifugation at 10,000 × *g* for 10 min at 4°C. Pellets were washed with 10 mM PBS (pH 7.4) and resuspended in the lysis buffer (8 M urea, 1% protease inhibitor). The resuspension was sonicated and the remaining debris was removed by centrifugation at 10,000 × *g* for 10 min at 4°C. The concentration of protein was determined with a BCA kit (Solarbio, China) after collecting supernatant. Detailed procedures of Trypsin digestion and LC-MS/MS analysis were described in the supplementary information (Text S1). All protein sequences were annotated using Uniprot (Release 2021_03), COG (updated in 2020), and KEGG databases (Release 99.1) (42). For comparative proteomic analyses, in the laboratorial condition, cells of E. flavus 21-3 cultivated in the medium supplemented with or without 40 mM sodium thiosulfate were respectively regarded as the active and silent sulfur-producing pathway; in the deep-sea *in situ* condition, E. flavus 21-3 WT and mutant Δ*soxB* were respectively regarded as the active and silent sulfur-producing pathway. Heatmap analysis and Venn diagram of studied proteins were completed by R packages pheatmap and VennDiagram respectively in R (v4.0.1).

### Metagenomic analysis.

Total DNA from 30 g sediments of each sample was extracted using Tianen Bacterial Genomic DNA Extraction Kit following the manufacturer’s protocol. Subsequent steps (metagenomic sequencing, assembly and binning) were shown in the supplementary information (Text S1). Genes used for analyzing sulfur cycle referred to the following research and were listed in Table S3 in the supplemental material (43). Sequences were annotated using KEGG (Release 87.0), NR (2021-10-17), uniport (Release 2021_03) and COG (updated in 2020) using Diamond (v0.8.23) with 1e-20 e-value cutoff (44). Heatmap analyses of studied genes were completed by R packages pheatmap in R (v4.0.1).

### Data deposit.

The genomic information of E. flavus 21-3 has been uploaded to the NCBI with the accession number CP032228. The proteomics data have been uploaded to Proteome Xchange Consortium with the data set identifier PXD016502 and PXD029383.

10.1128/mbio.00143-22.1 TEXT S1 Detailed procedures of proteomic profiling including trypsin digestion and LC-MS/MS analysis of peptides. Detailed description of metagenomic sequencing, assembly and binning. Download Text S1, DOCX file, 0.03 MB. Copyright © 2022 Cai et al. 2022 Cai et al. https://creativecommons.org/licenses/by/4.0/ This content is distributed under the terms of the Creative Commons Attribution 4.0 International license.
