# Supplementary material for: Deep-Sea In Situ Insights into the Formation of Zero-Valent Sulfur Driven by a Bacterial Thiosulfate Oxidation Pathway
Source: mBio. 2022 Jul 19;13(4):e00143-22. doi: 10.1128/mbio.00143-22 (PMC9426585; doi:10.1128/mbio.00143-22)
Supplement: FIG S1 [file mbio.00143-22-s0002.docx]

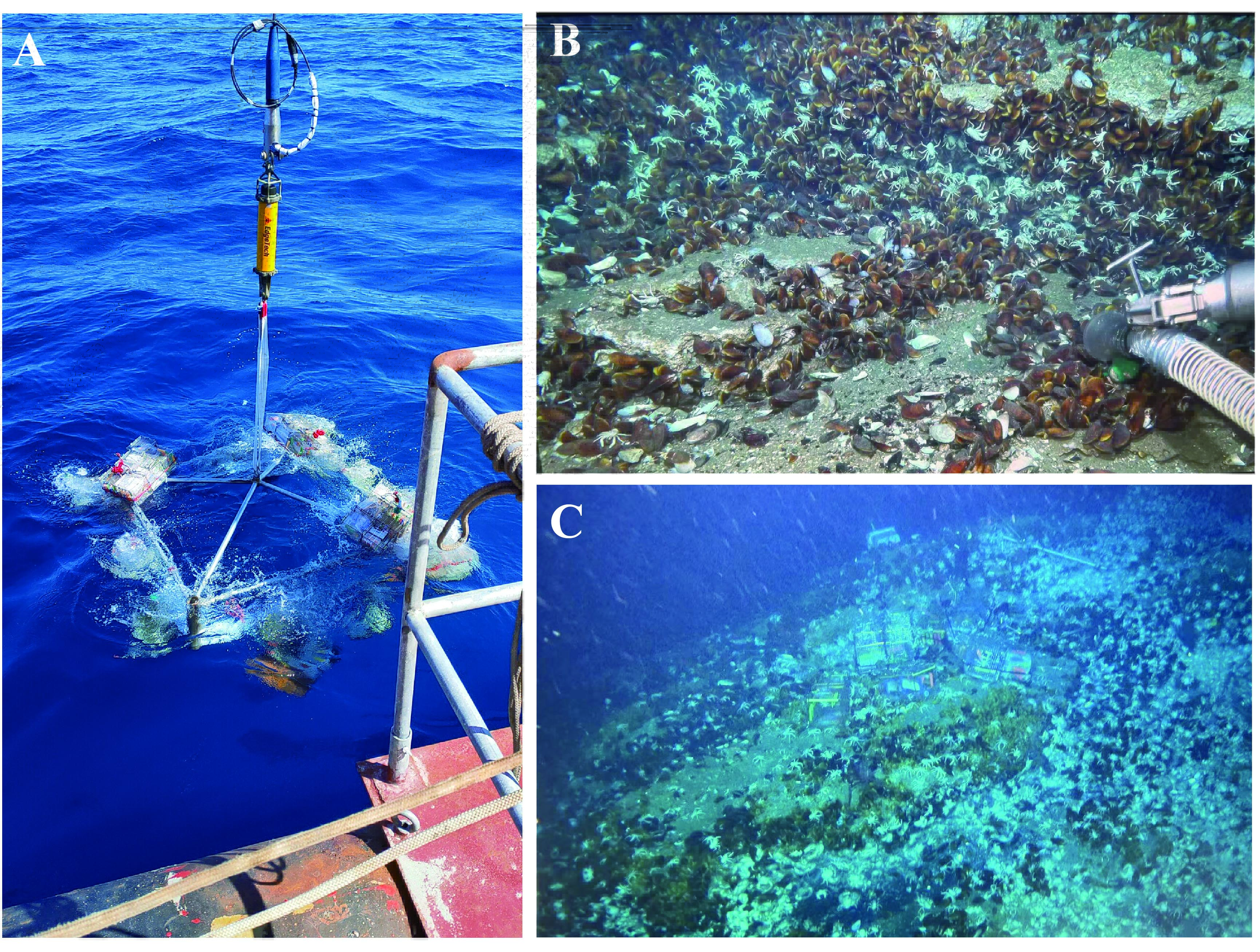


**Fig. S1. *In-situ* photos of the study process and site.** **(A)** The underwater remotely operated vehicle (*ROV*) was diving into the sea with samples. **(B, C)** *In-situ* photos of the deep-sea cold seep and the study process.
