# Supplementary material for: Deep-Sea In Situ Insights into the Formation of Zero-Valent Sulfur Driven by a Bacterial Thiosulfate Oxidation Pathway
Source: mBio. 2022 Jul 19;13(4):e00143-22. doi: 10.1128/mbio.00143-22 (PMC9426585; doi:10.1128/mbio.00143-22)
Supplement: TABLE S1 [file mbio.00143-22-s0005.docx]

**Table S1. The concentrations of sulfide, thiosulfate, sulfate and sulfite present in the seawater and sediments of the study sites**

|  | **Seawater** | **Sediments** |
| --- | --- | --- |
| Sulfide | 0.33 mM | 11.19 mM |
| Thiosulfate | Below limit of detection | 137.76 μM |
| Sulfate | 31.25 mM | 15.60 mM |
| Sulfite | Below limit of detection | Below limit of detection |

*Limit of detection: sulfite 3 μM, thiosulfate:4 μM.
