# Supplementary material for: Deep-Sea In Situ Insights into the Formation of Zero-Valent Sulfur Driven by a Bacterial Thiosulfate Oxidation Pathway
Source: mBio. 2022 Jul 19;13(4):e00143-22. doi: 10.1128/mbio.00143-22 (PMC9426585; doi:10.1128/mbio.00143-22)
Supplement: FIG S2 [file mbio.00143-22-s0003.docx]

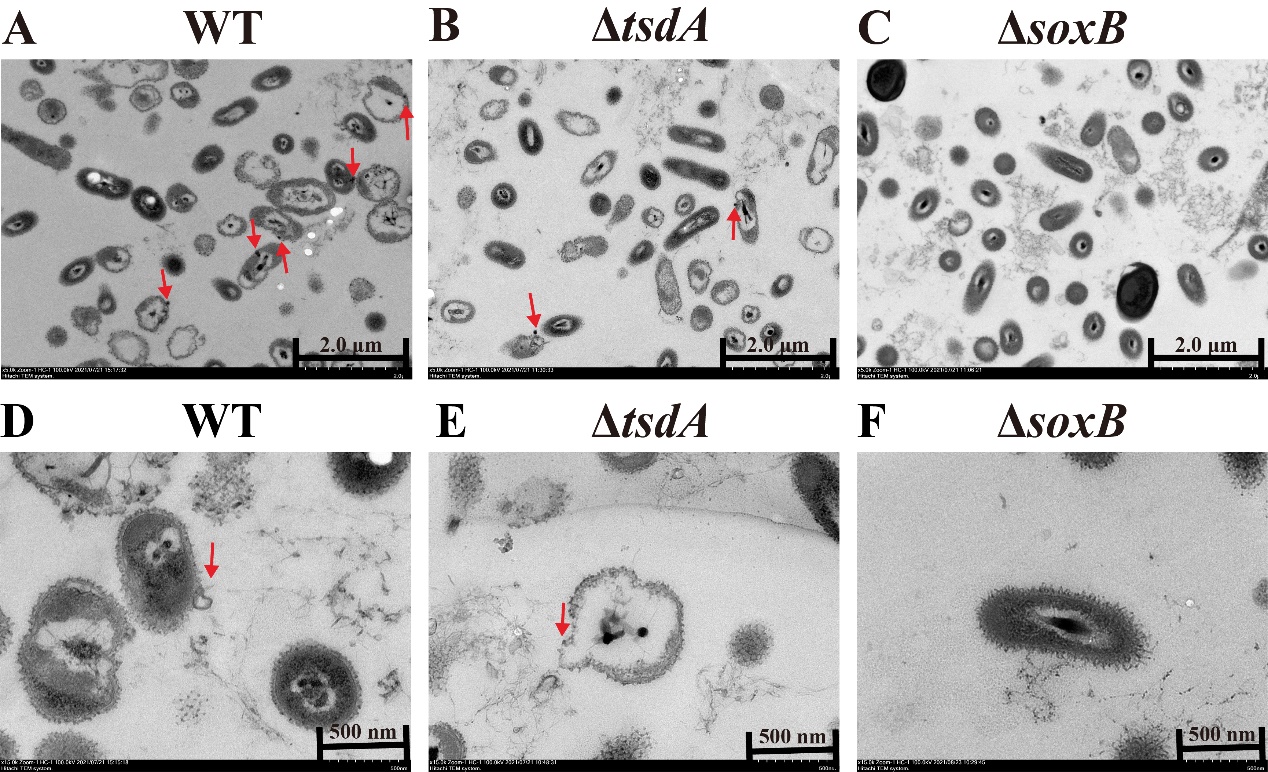


**Fig. S2** **TEM images of embedded and thin sectioned cells of *E. flavus* 21-3 wild type and mutants Δ*tsdA* and Δ*soxB* after 10-day *in-situ* incubation**. **(A-C)** Arrows indicate particles attaching to cells. **(D-F)** Arrows indicate the “membrane structure” attaching to cells.
