# Supplementary material for: Deep-Sea In Situ Insights into the Formation of Zero-Valent Sulfur Driven by a Bacterial Thiosulfate Oxidation Pathway
Source: mBio. 2022 Jul 19;13(4):e00143-22. doi: 10.1128/mbio.00143-22 (PMC9426585; doi:10.1128/mbio.00143-22)
Supplement: FIG S3 [file mbio.00143-22-s0004.docx]

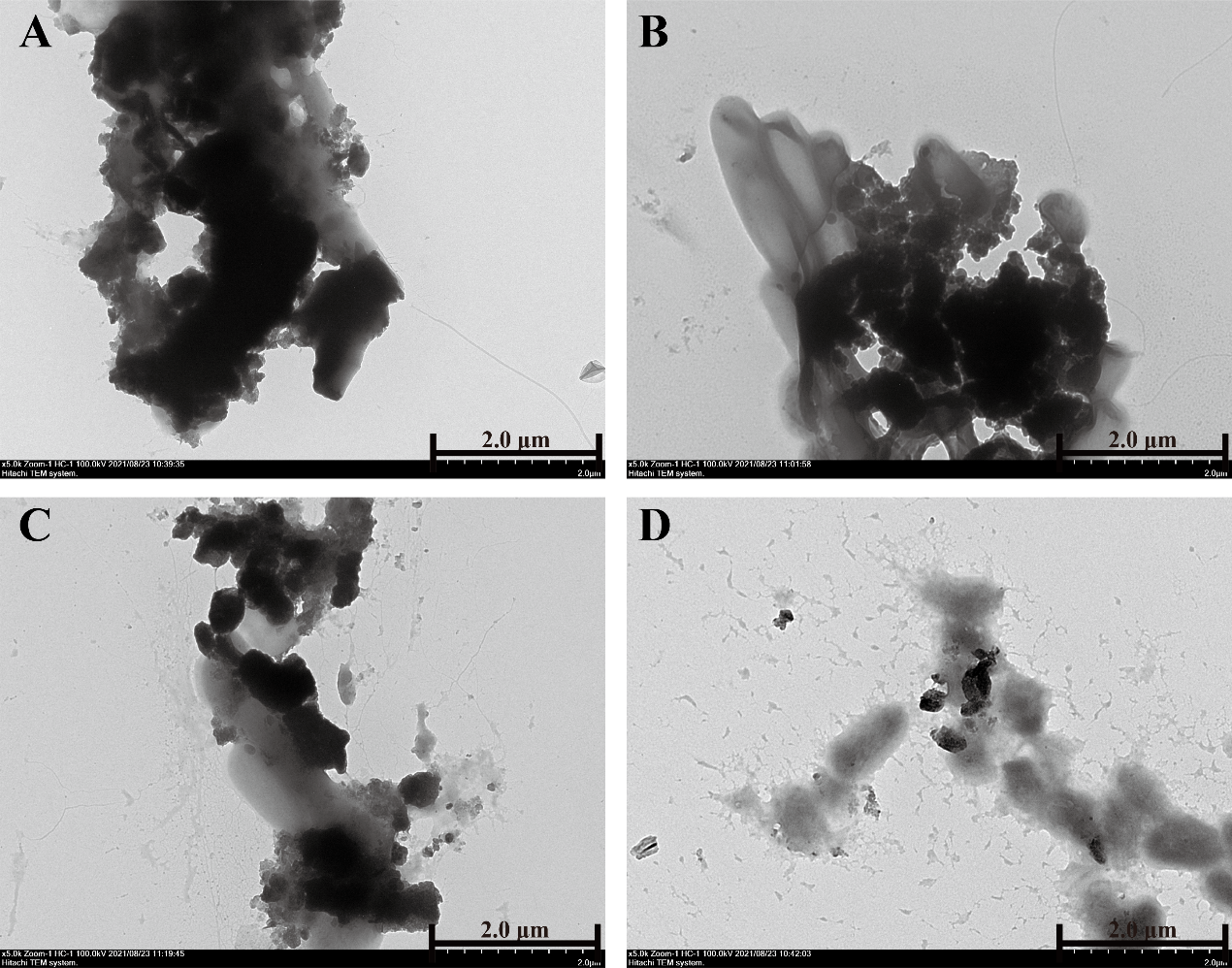


**Fig. S3** **TEM images of *E. flavus* 21-3 wild type that cultivated with sulfur globules as the sole electron donor after 7 days and 14 days**. **(A, B)** TEM images of *E. flavus* 21-3 wild type cultivated with ZVS after 7 days. **(C, D)** TEM images of *E. flavus* 21-3 wild type cultivated with ZVS after 14 days.
