# Supplementary material for: Deep-Sea In Situ Insights into the Formation of Zero-Valent Sulfur Driven by a Bacterial Thiosulfate Oxidation Pathway
Source: mBio. 2022 Jul 19;13(4):e00143-22. doi: 10.1128/mbio.00143-22 (PMC9426585; doi:10.1128/mbio.00143-22)
Supplement: TABLE S3 [file mbio.00143-22-s0007.docx]

**Table S3. Sulfur metabolism-related genes used in metagenomic analysis.**

| **Pathway** | **Enzyme** | **Gene ID** | **Gene name** |
| --- | --- | --- | --- |
| aryl-sulfate to sulfate | arylsulfatase | K01130 | *aslA* |
| DMS to DMSO | dimethyl sulfide dehydrogenase subunit alpha | K16964 | *ddhA* |
| sulfite oxidation | sulfite dehydrogenase (cytochrome) subunit B | K00386 | *sorB* |
| reversible reduction  of the heterodisulfide | heterodisulfide reductase subunit C | AEK59000.1 | *hdrC* |
| sulfite reduction | dissimilatory sulfite reductase alpha subunit  dissimilatory sulfite reductase beta subunit | K11180  K11181 | *dsrAB* |
| sulfide oxidation | sulfide: quinone oxidoreductase | K17218 | *sqr* |
| methanethiol oxidation | methanethiol oxidase | K17285 | *mtoX* |
| methionine to DMS | methionine-gamma-lyase | K01761 | *mgl* |
| MSA oxidation | methanesulfonate monooxygenase subunit alpha | K16968 | *msmA* |
| polysulfide reduction | polysulfide reductase chain A | K08352 | *psrA* |
| polysulfide reduction | sulfhydrogenase subunit beta | K17996 | *hydB* |
| polysulfide reduction | sulfhydrogenase subunit alpha delta gamma | K17993  K17994  K17995 | *hydDAG* |
| polysulfide reduction | NADH-dependent persulfide reductase [flavoprotein/rhodanase] | A3QAV3 | *npsr_rho* |
| polysulfide reduction | NADH-dependent persulfide reductase | Ga0364475_1298991618 | *npsr_Mod* |
| sulfate ester | choline-sulfatase | K01133 | *betC* |
| sulfate reduction | adenylylsulfate reductase, subunit A  adenylylsulfate reductase, subunit B | K00394  K00395 | *aprAB* |
| sulfate reduction | quinone-modifying oxidoreductase, subunit QmoA  quinone-modifying oxidoreductase, subunit QmoB  quinone-modifying oxidoreductase, subunit QmoC | K16885  K16886  K16887 | *qmo* |
| sulfate reduction | sulfate adenylyltransferase | K00958 | *sat* |
| sulfide oxidation | sulfide dehydrogenase  [flavocytochrome c] | K17229 | *fccB* |
| sulfide oxidation | sulfite dehydrogenase (quinone) subunit SoeA  sulfite dehydrogenase (quinone) subunit SoeB | K21307  K21308 | *soeAB* |
| sulfite oxidation | sulfite oxidase | K00387 | *suox* |
| sulfite reduction | anaerobic sulfite reductase | K16950  K16951 | *asrAB* |
| sulfonate | D-cysteine desulfhydrase | K05396 | *dcyD* |
| sulfonate | L-cysteate sulfo-lyase | K17950 | *cuyz* |
| sulfonate | sulfopropanediol 3-dehydrogenase | K15509 | *hspN* |
| sulfonate | taurine transport system substrate-binding protein | K15551 | *tauA* |
| sulfonate | sulfoacetaldehyde acetyltransferase | K03852 | *xsc* |
| sulfonium | dimethylpropiothetin dethiomethylase | K16953 | *dddL* |
| sulfonium | dimethylsulfoniopropionate demethylase | K17486 | *dmdA* |
| sulfonium | anaerobic dimethyl sulfoxide reductase subunit A  anaerobic dimethyl sulfoxide reductase subunit B  anaerobic dimethyl sulfoxide reductase subunit C | K07306  K07307  K07308 | *dmsABC* |
| sulfur transfer | tRNA 2-thiouridine synthesizing protein A | K04085 | *tusA* |
| tetrathionate reduction | tetrathionate reductase subunit A  tetrathionate reductase subunit B  tetrathionate reductase subunit C | K08357  K08358  K08359 | *ttrABC* |
| tetrathionate reduction | octaheme tetrathionate reductase | Q8E9W8 | *otr* |
| thiosulfate oxidation | thiosulfate sulfurtransferase  thiosulfate/3-mercaptopyruvate sulfurtransferase | K02439  K01011 | *glpE*  *TST* |
| thiosulfate oxidation | persulfide dioxygenase rhodanese | O95571 | *pdo_rho* |
| thiosulfate oxidation to  tetrathionate | thiosulfate dehydrogenase [quinone] large subunit | K16937 | *doxD* |
| thiosulfate oxidation to  tetrathionate | thiosulfate dehydrogenase | K19713 | *tsdA* |
| thiosulfate/polysulfide reduction | thiosulfate reductase cytochrome b subunit  thiosulfate reductase electron transport protein | K08353  K08354 | *phsBC* |
| thiosulfate/sulfide oxidation | sulfur-oxidizing complex | K17222  K17223  K17224  K17225  K17226  K17227 | *soxABCXYZ* |

*Gene list was referred to following research (1).

**References related to this table：**

1. Vigneron A, Cruaud P, Culley AI, Couture RM, Lovejoy C, Vincent WF. 2021. Genomic evidence for sulfur intermediates as new biogeochemical hubs in a model aquatic microbial ecosystem. Microbiome 9:1-14.
