# Supplementary material for: Deep-Sea In Situ Insights into the Formation of Zero-Valent Sulfur Driven by a Bacterial Thiosulfate Oxidation Pathway
Source: mBio. 2022 Jul 19;13(4):e00143-22. doi: 10.1128/mbio.00143-22 (PMC9426585; doi:10.1128/mbio.00143-22)
Supplement: TABLE S2 [file mbio.00143-22-s0006.docx]

**Table S2. Strains, plasmids and primers used in this study**

|  | **Relevant genotype, description or sequence** | **Reference or source** |
| --- | --- | --- |
| **Strains** |  |  |
| *Erythrobacter flavus* 21-3 | wild type, isolated from sediment in deep-sea clod seep | (1) |
| *E. flavus* 21-3 *ΔtsdA* | Deletion of *tsdA* (D0Y83_01395) in *E. flavus* 21-3 | (1) |
| *E. flavus* 21-3 *ΔsoxB* | Deletion of *soxB* (D0Y83_01365) in *E. flavus* 21-3 | (1) |
| **Plasmids** |  |  |
| pEX18Gm | 5.8 kb plasmid, Gm^R^, suicide vector | (2) |
| pEX-18Gm-KO-tsdA | Gm^R^, an overlap of 1750 bp inserted into pEX18Gm using *Eco*RⅠand *Bam*HⅠ | (1) |
| pEX-18Gm-KO-soxB | Gm^R^, an overlap of 1743 bp inserted into pEX18Gm using *Eco*RⅠand *Bam*HⅠ | (1) |
| **Primers (5’-3’)** |  |  |
| qRT-soxB-F | 5’-cctttgcctgctcgatgaga-3’ | this work |
| qRT-soxB-R | 5’-gtcggcgacagctatgaact-3’ | this work |
| qRT-tsdA-F | 5’-gaggtatggcgctgttcgta-3’ | this work |
| qRT-tsdA-R | 5’-ggcccttgtcgagatgacag-3’ | this work |
| qRT-16S-F | 5’-aaggccttcatcactcacgc-3’ | this work |
| qET-16S-R | 5’-cacactgggactgagacacg-3’ | this work |

**References related to this table：**

1. Zhang J, Liu R, Xi SC, Cai RN, Zhang X, Sun CM. 2020. A novel bacterial thiosulfate oxidation pathway provides a new clue about the formation of zero-valent sulfur in deep sea. Isme Journal 14:2261-2274.

2. Hoang TT, Karkhoff-Schweizer RR, Kutchma AJ, Schweizer HP. 1998. A broad-host-range Flp-FRT recombination system for site-specific excision of chromosomally-located DNA sequences: application for isolation of unmarked Pseudomonas aeruginosa mutants. Gene 212:77-86.
