# Supplementary material for: Deep-Sea In Situ Insights into the Formation of Zero-Valent Sulfur Driven by a Bacterial Thiosulfate Oxidation Pathway
Source: mBio. 2022 Jul 19;13(4):e00143-22. doi: 10.1128/mbio.00143-22 (PMC9426585; doi:10.1128/mbio.00143-22)
Supplement: TEXT S1 [file mbio.00143-22-s0001.docx]

**Supplementary Methods**

**Proteomic profiling: Trypsin digestion and LC-MS/MS analysis of peptides.** An equal amount of each sample protein for enzymatic hydrolysis was adjusted to the same volume with the lysing solution. Then, 1 volume of pre-cooled acetone and 4 volumes of pre-cooled acetone were added into protein samples and precipitated at -20°C for 20 min. Precipitates were collected by centrifugation at 4500 × *g* for 5 min and washed twice with pre-cooled acetone. After drying precipitates, TEAB was added with a final concentration of 200 mM. Suspensions were ultrasonically dispersed, mixed with trypsin solution at a ratio of 1:50 (protease: protein, m/m) and hydrolyzed overnight. Then, dithiothreitol (DTT) was added to a final concentration of 5 mM and the mixture was reduced at 20°C for 30 min. Finally, iodoacetamide (IAA) was added to make the final concentration 11 mM and incubated for 15 min at room temperature in dark.

Tryptic peptides were separated by the ultra-performance liquid chromatography (UPLC) and ionized by NSI ion source and then analyzed by Orbitrap Exploris™ 480 mass spectrometer. The voltage of the ion source was 2.3 kV and the compensation voltage of FAIMS was -45 V and -65 V. Peptide precursor ions and their secondary fragments were detected and analyzed by high-resolution Orbitrap. The scanning range of the primary mass spectrum was 400-1200 m/z, the scanning resolution was 60000, the fixed starting point of the scanning range of the secondary mass spectrum was 110 m/z and the secondary scanning resolution was 15000. The data acquisition mode was the Cycle time-based data-dependent scanning (DDA) program. To improve the effective utilization of the mass spectrometer, the automatic gain control (AGC) was set as100%, the signal threshold was 5E4 ions/s, the maximum injection time was Auto, and the dynamic rejection time of the tandem mass spectrometry scan was 20 s to avoid scanning precursor ions repeatedly.

**Metagenomic sequencing, assembly and binning.** Total DNA from 30 g sediments of each sample was extracted using the Tianen Bacterial Genomic DNA Extraction Kit following the manufacturer’s protocol. Extracts were treated with DNase-free RNase to eliminate RNA contamination. DNA concentration was measured using a Qubit 3.0 fluorimeter. Then, DNA integrity was evaluated by gel electrophoresis and 0.5 μg of each sample was used to prepare libraries. DNA was sheared into fragments between 50 ~ 800 bp using the Covaris E220 ultrasonicator (Covaris, Brighton, UK). DNA fragments between 150 ~ 250 bp were selected using AMPure XP beads (Agencourt, Beverly, MA, USA) and then were repaired using T4 DNA polymerase (ENZYMATICS, Beverly, MA, USA). These DNA fragments were ligated at both ends to T-tailed adapters and amplified for eight cycles. Finally, amplification products were subjected to a single-strand circular DNA library.

All NGS libraries were sequenced on the BGISEQ-500 platform (BGI-Qingdao, China) to obtain 100 bp paired-end raw reads. Quality control was performed by SOAPnuke (v1.5.6) (setting: -l 20 -q 0.2 -n 0.05 -Q 2 -d -c 0 -5 0 -7 1) (1). The clean data were assembled using MEGAHIT (v1.1.3) (setting:--min-count 2 --k-min 33 --k-max 83 --k-step 10) (2). Thereafter, metaBAT2 (3), Maxbin2 (4) and Concoct (5) were used to automatically bin from assemblies. Finally, MetaWRAP (6) was used to purify and arrange data into final bins. Completeness and contamination were calculated by checkM (v1.0.18) (7).

**References related to these methods:**

1. Chen YX, Chen YS, Shi CM, Huang ZB, Zhang Y, Li SK, Li Y, Ye J, Yu C, Li Z, Zhang XQ, Wang J, Yang HM, Fang L, Chen Q. 2017. SOAPnuke: a MapReduce acceleration-supported software for integrated quality control and preprocessing of high-throughput sequencing data. Gigascience 7:1-6.

2. Li DH, Liu CM, Luo RB, Sadakane K, Lam TW. 2015. MEGAHIT: an ultra-fast single-node solution for large and complex metagenomics assembly via succinct de Bruijn graph. Bioinformatics 31:1674-1676.

3. Kang DWD, Li F, Kirton E, Thomas A, Egan R, An H, Wang Z. 2019. MetaBAT 2: an adaptive binning algorithm for robust and efficient genome reconstruction from metagenome assemblies. Peerj 7.

4. Wu YW, Simmons BA, Singer SW. 2016. MaxBin 2.0: an automated binning algorithm to recover genomes from multiple metagenomic datasets. Bioinformatics 32:605-607.

5. Alneberg J, Bjarnason BS, de Bruijn I, Schirmer M, Quick J, Ijaz UZ, Lahti L, Loman NJ, Andersson AF, Quince C. 2014. Binning metagenomic contigs by coverage and composition. Nature Methods 11:1144-1146.

6. Uritskiy GV, DiRuggiero J, Taylor J. 2018. MetaWRAP-a flexible pipeline for genome-resolved metagenomic data analysis. Microbiome 6.

7. Parks DH, Imelfort M, Skennerton CT, Hugenholtz P, Tyson GW. 2015. CheckM: assessing the quality of microbial genomes recovered from isolates, single cells, and metagenomes. Genome Research 25:1043-1055.
